# Highly Efficient Capacitive Deionization Enabled by NiCo_4_MnO_8.5_ Electrodes

**DOI:** 10.1002/gch2.202100095

**Published:** 2021-11-16

**Authors:** Wei Wang, Zhenzhen Liu, Zehao Zhang, Haibo Li

**Affiliations:** ^1^ Ningxia Key Laboratory of Photovoltaic Materials Ningxia University Yinchuan Ningxia 750021 China

**Keywords:** capacitive deionization, desalination, ternary metal oxides

## Abstract

The shortage of fresh water resources is one of the major challenges facing this planet. Capacitive deionization (CDI) techniques that are deemed to be highly efficient and require low capital cost have attracted widespread attention in the last few decades. In this work, the cubic ternary metal oxides NiCo_4_MnO_8.5_ (Ni–Co–Mn–O) are synthesized by facile hydrothermal method for enhanced symmetrical CDI. Electrochemical measurements illustrate that the Ni–Co–Mn–O possesses low internal resistance and ion diffusion impedance. As a result, the salt removal capacity of the Ni–Co–Mn–O electrode increases from 26.84 to 65.61 mg g^−1^ by varying the voltage from 0.8 to 1.4 V in 1.0 × 10^−2^ m NaCl solution, while the charge efficiency stabilizes at ≈80%. After 20 cycles, the capacitance retained is 64.27%, which is due to the irreversibility of Co^2+^/Co^3+^ and Mn^2+^/Mn^3+^ and the release of Ni^3+^ from the Ni–Co–Mn–O electrode after long desalination/salination cycles.

## Graphical abstract

A ternary metal oxide NiCo_4_MnO_8.5_ electrode is proposed for capacitive desalination. Excellent desalination performance is achieved, including high salt removal capacity, long cycling stability, and high charge efficiency. In addition, the corresponding desalination mechanism is explored.

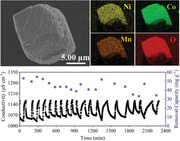

## 1 Introduction

Fresh water resources are one of the main ecological issues to determine the development of human society. Unfortunately, the explosive growth of population and the rapid development of economy lead to the issue of portable water shortage.^[^ ^1^, ^2^, ^3^ ^]^ On the other hand, 70% of the Earth's surface is covered by the sea water which provides the great potential to address this problem.^[^ ^4^, ^5^ ^]^ Capacitive deionization (CDI) is a newly developed technique for microbrine desalination that has the advantages of high efficiency and low cost over the traditional desalination technologies.^[^ ^1^, ^2^, ^6^, ^7^ ^]^ The typical CDI systems is operated at low voltages which forces the cation and anion to be held onto the interfaces between the electrodes and solution, resulting in the purpose of desalination.^[^ ^8^ ^]^ In the past few decades, the carbon‐based electrodes are very popular in application to CDI including activated carbon, carbon nanotubes, graphene, etc.^[^ ^8^, ^9^, ^10^, ^11^, ^12^ ^]^ Despite the carbon electrodes own the huge specific surface area, special microporous structure and low fabrication cost, it merely relies on physical adsorption, which cause low desalination capacity and current efficiency in high concentrated brine.^[^ ^10^, ^13^, ^14^ ^]^

Recently, the Faradaic reactions electrodes have demonstrated remarkable salt removal capacity and current efficiency in CDI research since they can store ions within the crystal structures.^[^ ^15^, ^16^, ^17^, ^18^, ^19^, ^20^ ^]^ Among all Faradaic electrodes, the transition metal oxides have drawn great attentions in energy storage research field due to their suitability in aqueous solution,^[^ ^15^, ^16^, ^21^ ^]^ i.e., NiO, Co_3_O_4_, and MnO_2_.^[^ ^22^ ^]^ Nevertheless, the low conductivity and ion diffusion rate associated with transition metal oxides are harmful to their electrochemical activity.^[^ ^23^ ^]^ In order to overcome this issue, the ternary metal oxides (TMOs) are proposed, which utilizes the synergistic effect originating from different metal ions, resulting in the improved electrical conductivity and diffusion rate.^[^ ^24^ ^]^ Moreover, the redox potentials of transition metal oxides can be tunneled by controlling the kinds and amount of metal elements.^[^ ^21^, ^25^ ^]^ In the family of transition metal oxides, the TMOs have multiple oxidation states which enable higher ions storage capacity than single component metal oxides as well as multiple redox reactions during the charging/discharging.^[^ ^25^, ^26^ ^]^

In this work, we synthesized the spinel NiCo_4_MnO_8.5_ (Ni–Co–Mn–O) by hydrothermal method for CDI with enhanced desalination capacity. The morphology, structure and electrochemical behavior of Ni–Co–Mn–O are explored. Further, the desalination performance of Ni–Co–Mn–O is carried out employing batch‐mode CDI system in 500 ppm NaCl solution, such as capacity, rate, and cyclic ability, as well as the corresponding desalination mechanism.

## 2 Results and Discussion

**Figure 1:**
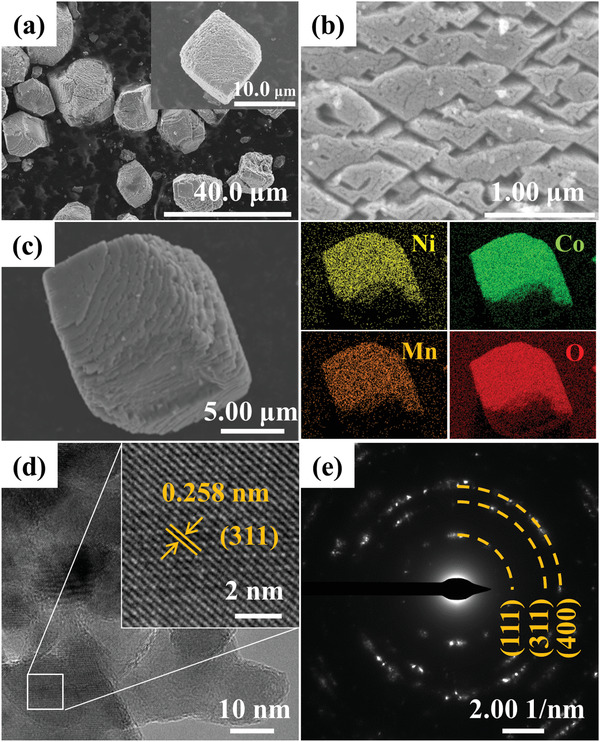
SEM images of the Ni–Co–Mn–O in low a) and high magnifications b,c) and elemental mapping images, the d) HRTEM image, and e) SAED pattern of Ni–Co–Mn–O.

**Figure** **1**a shows the scanning electron microscopy (SEM, Hitachi SU5000, Japan) image of Ni–Co–Mn–O which present typical micrometer‐sized particle with irregular geometry. The magnified SEM image of Ni–Co–Mn–O in inset of Figure 1a as well as Figure 1b exhibit a stacked layered structure. Apparently, it is observed from Figure 1b that a plenty of holes are randomly distributing on the surface of Ni–Co–Mn–O, which is deductive to promote the diffusion of salty ions and thus the kinetics of desalination. Figure 1c exhibits the surface element distribution of Ni–Co–Mn–O which was confirmed by the energy dispersive spectrometer (EDS). The Ni (1.33%), Co (23.35%), Mn (3.08%), and O (72.24%) demonstrate the even distribution. Figure 1d shows the high resolution transmission electron microscopy (HRTEM, FEI Talos200s, USA) image of the Ni–Co–Mn–O where the inset image realizes an clear lattice fringe with spacing of 0.258 nm corresponding to the (311) plane of the Ni–Co–Mn–O. Beyond that, the selected areal electron diffraction (SAED) image of Ni–Co–Mn–O illuminates regular a set of diffraction rings which are consistent with the lattice planes of (111), (311), and (400), respectively.

**Figure 2:**
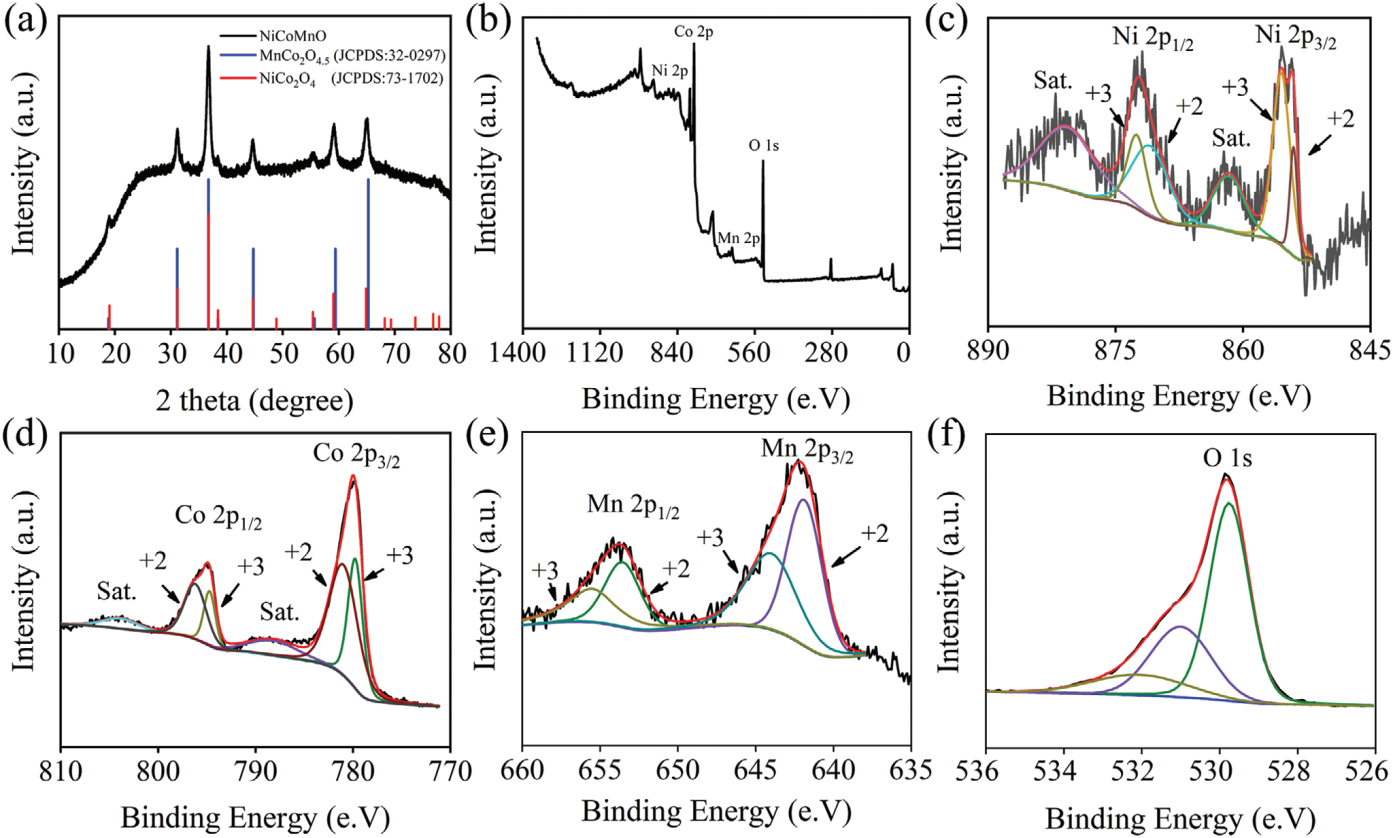
a) XRD pattern of Ni–Co–Mn–O, b) survey, c) Ni 2p, d) Co 2p, e) Mn 2p, and f) O 1s spectrum of Ni–Co–Mn–O.

The phase analysis of Ni–Co–Mn–O was performed on the X‐ray diffraction (XRD, SmartLab, Japan) in **Figure** **2**a, revealing the prefect spinel structure of Ni–Co–Mn–O.^[^ ^27^ ^]^ Further, the XRD pattern of Ni–Co–Mn–O exhibit nine distinct characteristic diffraction peaks which matches to the MnCo_2_O_4.5_ and NiCo_2_O_4_ crystals phase very well (JCPDS card #32‐0297 and #73‐1702). The X‐ray photoelectron spectroscopy (XPS, Thermo ESCALAB 250Xi) was employed to explore the valence states of element in Ni–Co–Mn–O in Figure 2b. As shown in Figure 2c in relation to the Ni 2p spectrum, two peaks near to 855.4 and 871.9 eV are in terms of Ni 2p_3/2_ and Ni 2p_1/2_, accompanied by two satellite peaks, respectively. Further, the subpeaks at ≈854.1 and 870.9 eV are caused by the electron transitions in the outer layer of Ni^2+^, while the subpeaks at ≈855.5 and 872.5 eV are attributed to the presence of Ni^3+^.^[^ ^28^ ^]^ Figure 2d draws the Co 2p spectrum. It displays two peaks at 779.9 and 795.2 eV which are assigned to the Co 2p_3/2_ and Co 2p_1/2_ with two satellite peaks, respectively. The subpeaks related to Co^2+^ are located at ≈780.9 and 796.2 eV, while the subpeaks associated with Co^3+^ are captured at ≈779.8 and 794.7 eV.^[^ ^29^, ^30^ ^]^ Figure 2e shows the Mn 2p spectrum, evidencing two characteristic peaks at 642.3 and 654.2 eV which correspond to Mn 2p_3/2_ and Mn 2p_1/2_, respectively. The subpeaks at ≈653.5 and 641.8 eV can be attributed to the presence of Mn^2+^, while the subpeaks at ≈655.5 and 643.9 eV are related to Mn^3+^.^[^ ^30^, ^31^ ^]^ The spectrum of O 1s (Figure 2f) reveals the electron states of three kinds of oxygen. Specifically, the peak at 529.4 eV represents the metal‐oxygen bonds while the peak at 531.2 eV is usually associated with the surface species, i.e., hydroxyls. Moreover, the peak at 532.5 eV may be related to water adsorbed on the surface of the Ni–Co–Mn–O.^[^ ^31^ ^]^ Based on the XPS analysis, the abundant valance states enabled by Co, Mn, and Ni can generate multiredox couples which are beneficial to promote the desalination capacity.

**Figure 3:**
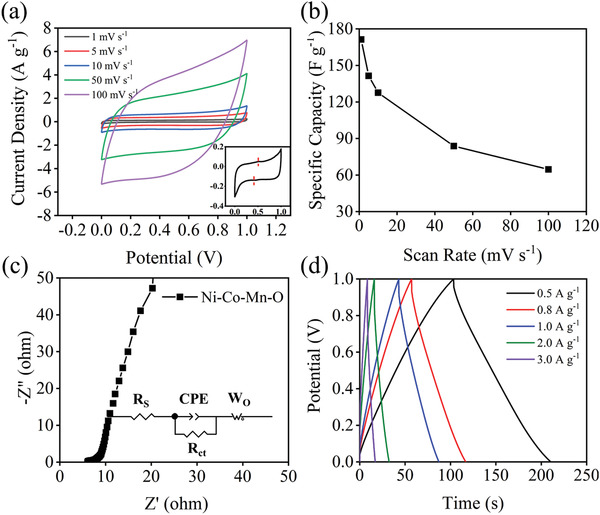
a) *CV* curve of Ni–Co–Mn–O electrode and the inset shows an enlarged *CV* curve at 1 mV s^−1^, b) corresponding specific capacitance with respect to scanning rates, c) EIS curve, and d) GCD at different rates.

The electrochemical behaviors of Ni–Co–Mn–O were carried out on the electrochemical workstation (CHI 660D, China) via three‐electrode system including cyclic voltammetry (CV), electrochemical impedance spectroscopy (EIS), and galvanostatic charging/discharging (GCD). **Figure** **3**a shows the CV curves of Ni–Co–Mn–O at different scanning rates in 1 m NaCl with potential varying from 0 to 1.0 V. Basically, they exhibit pseudorectangular shape. The redox peak can be clearly captured in the inset of Figure 3a. The area of CV curve is depending on the scan rate. Accordingly, the specific capacitance is illuminated in Figure 3b. It can be calculated by $C\;=\frac{\int I\;dV}{m\nu \Delta \;\;V}$, where *I*, *m*, *v*, and Δ*V* represent the current (A), the mass of the active material (g), scan rate (mV s^−1^), and the potential window (V), respectively. Obviously, the specific capacitance decreases with the increase of scanning rate, and the maximum specific capacitance is 171.30 F g^−1^ at 1 mV s^−1^. Figure 3c shows the EIS from 0.01 to 10^6^ Hz. Theoretically, the horizontal intercept of the Z′ axis represents the total internal resistance (*R*_S_) of the equivalent series circuit. The slope of the line is the Warburg impedance (*W*_O_), which corresponds to the diffusion impedance of amphoteric ions at the electrode/electrolyte interface. The EIS curve indicates that the Ni–Co–Mn–O has a small horizontal intercept in the low frequency region and a large slope in the high frequency region, which suggests that the Ni–Co–Mn–O has a low internal resistance and ion diffusion impedance. According to simulation, the *R*_S_ and *W*_O_ is 6.09 and 4.65 Ω, respectively. Figure 3d shows the GCD curve at different charging rates, demonstrating good symmetry at different magnification rates, which implies that the Ni–Co–Mn–O has excellent charge/discharge cyclic ability.

Prior to experiment, the CDI electrode was prepared according to the method that descripted in our previous report.^[^ ^32^ ^]^ Basically, the as‐prepared Ni–Co–Mn–O powder was mixed with conductive carbon black and PVDF according to the mass ratio of 8:1:1. Then, the mixture was shifted into an agate mortar to grind completely, accompanied by adding an appropriate amount of *N*‐methyl‐2‐pyrrolidone. After that, the resulting mixture is evenly coated on a 6 cm × 6 cm graphite sheet. Finally, the electrode was placed in an oven and dried at 60 °C for 12 h. In this experiment, two identical graphite sheets with the effective dimension of 5 cm × 5 cm were selected to adopt the Ni–Co–Mn–O for CDI electrodes operating in batch mode.

**Figure 4:**
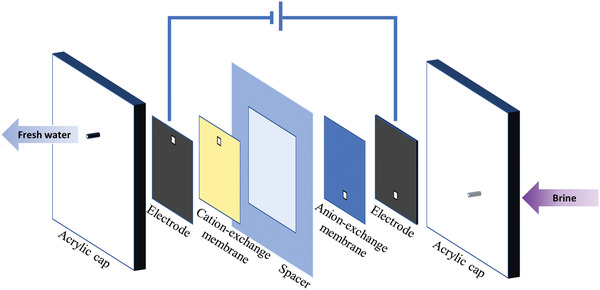
The schematic of CDI cell.

**Figure** **4** draws the schematics of CDI unit. Two pieces of acrylic caps were used for external fixation, and the electrodes were separated by a spacer to avoid short circuit. Notably, the ion‐exchange membrane was placed in front of active electrode to minimize the negative impact of co‐ions. Further, 40 mL NaCl solution with an initial conductivity of 1160 µS cm^−1^ (≈1.0 × 10^−2^ m) was employed as electrolyte. In typical desalination experiment, a fixed voltage (0.8, 1.0, 1.2, and 1.4 V) was applied on the CDI module for 60 min to achieve the desalination. Afterward, –0.8 V was introduced to both electrodes to complete the regeneration. Notably, the real‐time conductivity and current were recorded independently during the desalination. The salt removal capacity (*Γ*, mg g^−1^) is calculated by Equation (1)

(1) $$\begin{array}{c} \Delta \;\;=\frac{\left(\sigma_{0}-\sigma \right)\;\times V}{m} \end{array}$$

where σ_0_ and σ are the initial concentration (mg L^−1^) and the concentration at the end of the desalination, *V* (L) is the volume of NaCl, and *m* is the total effective mass of two electrodes (g). In addition to *Γ*, the charge efficiency (*Λ*) is calculated from Equation (2)

(2) $$\begin{array}{c} \wedge \;=\frac{\Gamma \;\times F}{M\frac{\int i\;dt}{m}} \end{array}$$

where *F* is the Faraday constant (96 485 C g^−1^), *M* is the molar mass of NaCl (58.44 g mol^−1^), and *i* is the electric current (mA).

**Figure 5:**
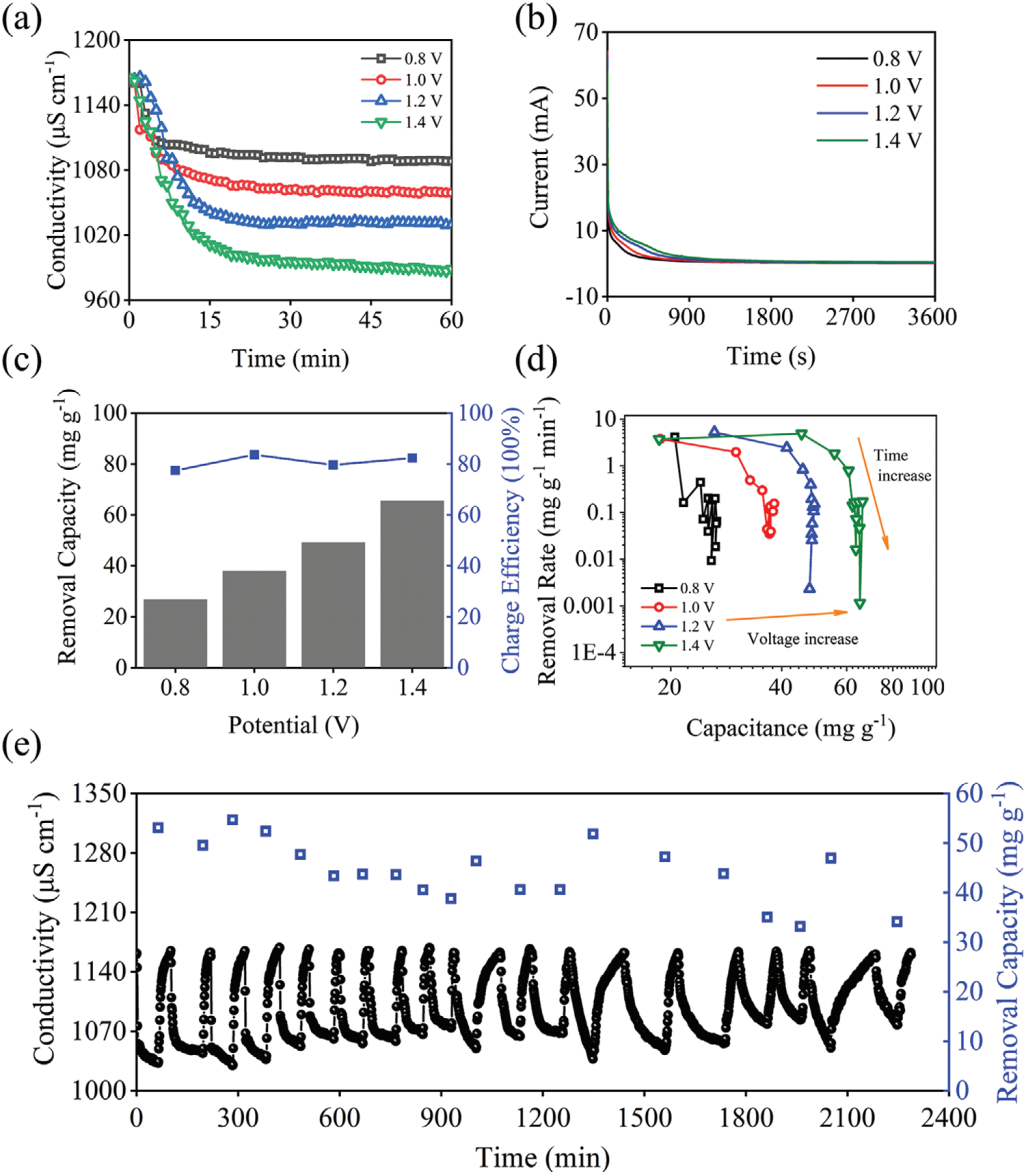
a) the real‐time conductivity of Ni–Co–Mn–O CDI, b) salt removal capacity and charge efficiency in terms of voltage, c) current variation of Ni–Co–Mn–O CDI, d) the CDI Ragone plot, and e) cycling performance.

The desalination performance of Ni–Co–Mn–O in 1160 µS cm^−1^ NaCl is displayed in **Figure** **5**a where the conductivity varied with time under 0.8 to 1.4 V. While Figure 5b shows corresponding current transient. At any voltage, the conductivity decrease rapidly in the initial stage due to the capture of Na^+^ and then become stable because of the saturation. Meanwhile, the current variation exhibits the similar trend with the conductivity. Further, the relationship between the *Γ* and voltage, together with the *Λ*, is given in Figure 5c. Upon the voltage varying from 0.8 to 1.4 V, the *Γ* increases from 26.84 to 65.61 mg g^−1^. Significantly, the *Λ* stabilize at around 80%, realizing high utilization of input energy. Figure 5d shows the effect of voltage on the Ragone plot in 1160 µS cm^−1^ NaCl solution. With the increase of voltage, the Ragone plot curve gradually moves to the upper right, which manifests that the increase of voltage leads to the increase the capacitance and removal rate of Ni–Co–Mn–O. Figure 5e shows the cycling performance of Ni–Co–Mn–O CDI in 1160 µS cm^−1^ NaCl at 1.2 V. As shown, the *Γ* is 53.09 mg g^−1^ at the first cycle. After 20 cycles, the *Γ* decrease to 34.12 mg g^−1^, illuminating the capacitance retention of 64.27%. In order to further demonstrate the superior desalting ability of Ni–Co–Mn–O electrode, **Table** **1** is provided which summarizes the desalination capability of different electrodes.

**Table 1:** Comparison of salt removal capacity among various CDI electrodes

Materials	Voltage [V]	Initial Con. [mg L^−1^]	Capacity [mg g^−1^]	Ref.
HCZ	1.4	250	55.2	^[^ ^33^ ^]^
MgAl‐Ox/G	1.0	500	13.6	^[^ ^34^ ^]^
Hybrid‐MnO_2_	1.2	870	27.3	^[^ ^35^ ^]^
Na_2_FeP_2_O_7_	1.2	580	30.2	^[^ ^36^ ^]^
Na_4_Mn_9_O_18_	1.2	580	31.2	^[^ ^37^ ^]^
CuAl‐LDO	1.2	500	39.1	^[^ ^38^ ^]^
NP‐3DHCA	1.2	500	26.8	^[^ ^39^ ^]^
NMCs‐800	1.2	584	20.6	^[^ ^40^ ^]^
NGCPs	1.4	500	20.1	^[^ ^41^ ^]^
Ni–Co–Mn–O	1.4	580	65.6	This work

**Figure 6:**
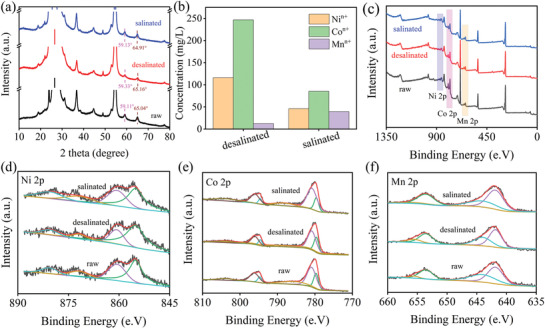
a) XRD patterns of raw, desalinated, and salinated electrode, b) ionic concentration of raw, desalinated, and salinated stream, c) XPS full survey spectra, d) Ni 2p, e) Co 2p, and f) Mn 2p spectrum of raw, desalinated, and salinated electrode.

The crystal phase characteristics of Ni–Co–Mn–O in different stage are explored in **Figure** **6**a. The peak at 59.11° and 65.04° shift to the right during the adsorption process, indicating the shrunken lattice spacing of (511) and (440) plane. In the desalination process, the Ni ions and Co ions are separated from the electrode to form vacancies, and Na ions enter the vacancy to reduce the lattice spacing. In the desorption process, the recovery of these diffraction peaks indicates that Na ions are exited and Ni ions and Co ions return to vacancy. In addition, the change of peak strength of other peaks also indicates that the lattice of Ni–Co–Mn–O has changed during this process. The ion concentration during the desalination and salination are monitored by the inductively coupled plasma mass spectrometry (ICP‐MS, Aglient 7800, USA) in Figure 6b. In desalination, the concentration of Ni, Co and Mn ions increases, while the concentration of Ni and Co ions recovered a lot excepting for Mn ions. This is probably accounted for the capacitance decline after long cycles. Figure 6c–f draws the full survey spectra, Ni 2p, Co 2p, and Mn 2p spectra of Ni–Co–Mn–O electrode in different state. In Figure 6c, the peak intensity of Co 2p and Ni 2p decreases during the desalination process, implying the Co and Ni ions escape from the crystal structure. By calculating, it is obtained that the ratio of Co^2+^/Co^3+^ decreased from 2.64 to 2.3 after the desalination. Actually, during the desalination, the reduction reaction relating to Co^2+^/Co^3+^ redox couple occurs on the electrode. However, a large number of Co^2+^ are released into the stream, leading to the rise of Co ions concentration and the decrease of the ratio of Co^2+^/Co^3+^ in Ni–Co–Mn–O electrode. In the meanwhile, the Mn^2+^/Mn^3+^ redox couple undergoes the reduction reaction as well, the ratio of Mn^2+^/Mn^3+^ increase from 1.77 to 1.83. In contrast to Co^2+^/Co^3+^ and Mn^2+^/Mn^3+^, a large portion of Ni ions are released, referring to Figure 6b. In salination process, the ratio of Co^2+^/Co^3+^ increase to 3.66 due to the reinsertion of Co^2+^. Meanwhile, the ratio of Mn^2+^/Mn^3+^ increase to 2.38 due to the release of Mn^3+^. Unfortunately, relatively high amount of Ni, Co and Mn ions cannot be recovered to the initial value which causes the capacitance decline as reflected in Figure 5e. Moreover, the raw, desalinated and salinated electrodes are characterized by the EDS. In the raw electrode, the atomic ratio of Ni, Co and Mn is 8.03%, 76.76%, and 15.21%, respectively, which change to 7.87%, 76.70%, and 15.43% correspondingly after desalination. After salination, they are separately 7.87%, 77.16%, and 14.96%.

## 3 Conclusions

In summary, the ternary metal oxide NiCo_4_MnO_8.5_ (Ni–Co–Mn–O) has been synthesized and proposed as high‐performance electrode for capacitive deionization (CDI). The Ni–Co–Mn–O presents typical micrometer‐sized particle with irregular geometry. It exhibits highly crystallinity which matches to the MnCo_2_O_4.5_ and NiCo_2_O_4_ crystals phase very well. The XPS spectra demonstrates the existence of Co^2+^/Co^3+^ and Mn^2+^/Mn^3+^ redox couple in Ni–Co–Mn–O. Further, the maximum specific capacitance of Ni–Co–Mn–O electrode is 171.30 F g^−1^ at 1 mV s^−1^ in 1 m NaCl solution. Toward the CDI, the desalination capacity of Ni–Co–Mn–O electrode is 49.11 mg g^−1^ at 1.2 V in 1160 µS cm^−1^ (≈1 × 10^−2^ m) NaCl solution. Beyond that, the mechanism analysis suggests that the irreversibility of Co^2+^/Co^3+^ and Mn^2+^/Mn^3+^ and the release of Ni^3+^ are accounted for the capacitance decay of Ni–Co–Mn–O electrode after long desalination/salination cycle.

## 4 Experimental Section

### Reagents

Ni(NO_3_)_2_·6H_2_O (Sinopharm Chemical Reagent), Co(NO_3_)_2_·6H_2_O (Macklin), Mn(NO_3_)_2_·4H_2_O (Aladdin), and urea (Sinopharm Chemical Reagent) were used as reagents. All chemicals are analytical grade reagents and have not undergone any purification treatment.

### Synthesis of Ni–Co–Mn–O

First, 0.6052 g Ni(NO_3_)_2_·6H_2_O, 2.4228 g Co(NO_3_)_2_·6H_2_O, and 0.5224 g Mn(NO_3_)_2_·4H_2_O were solved in 35 mL deionized water (DI) and labeled as solution A. 30.03 g urea was dissolved in 100 mL DI to form as solution B. Then, solution B was slowly added to solution A and stirred for 30 min. Subsequently, the 75 mL mixed solution was transferred into a 100 mL stainless‐steel autoclave lined with Teflon. The autoclave was sealed and maintained at 140 °C for 12 h in an electric oven. The product was then centrifuged 5 times and scrubbed 3 times with both DI and anhydrous ethanol. Afterward, it was dried in an oven at 80 °C for 3 h to obtain the precursor. Finally, the precursor was annealed at 400 °C for 3 h at a heating rate of 10 °C min^−1^ and cooled to room temperature in muffle furnace to obtain the Ni–Co–Mn–O.

## Conflict of Interest

The authors declare no conflict of interest.

## Data Availability

Research data are not shared.
